# Accuracy and stability of an arterial sensor for glucose monitoring in a porcine model using glucose clamp technique

**DOI:** 10.1038/s41598-020-63659-4

**Published:** 2020-04-20

**Authors:** Felix Aberer, Verena Theiler-Schwetz, Haris Ziko, Bettina Hausegger, Iris Wiederstein-Grasser, Daniel A. Hochfellner, Philipp Eller, Georg Tomberger, Martin Ellmerer, Julia K Mader, Vladimir Bubalo

**Affiliations:** 1 Medical University of Graz, Division of Endocrinology and Diabetology, Department of Internal Medicine, Graz, Austria; 2 Medical University of Graz, Core Facility Experimental Biomodels, Division of Biomedical Research, Graz, Austria; 3 Medical University of Graz, Intensive Care Unit, Department of Internal Medicine, Graz, Austria; 4 BBraun Melsungen AG, Research and Development Site Graz, Graz, Austria

**Keywords:** Preclinical research, Translational research, Diabetes

## Abstract

Intravascular glucose sensors have the potential to improve and facilitate glycemic control in critically ill patients and might overcome measurement delay and accuracy issues. This study investigated the accuracy and stability of a biosensor for arterial glucose monitoring tested in a hypo- and hyperglycemic clamp experiment in pigs. 12 sensors were tested over 5 consecutive days in 6 different pigs. Samples of sensor and reference measurement pairs were obtained every 15 minutes. 1337 pairs of glucose values (range 37–458 mg/dl) were available for analysis. The systems met ISO 15197:2013 criteria in 99.2% in total, 100% for glucose <100 mg/dl (n = 414) and 98.8% for glucose ≥100 mg/dl (n = 923). The mean absolute relative difference (MARD) during the entire glycemic range of all sensors was 4.3%. The MARDs within the hypoglycemic (<70 mg/dl), euglycemic (≥70–180 mg/dl) and hyperglycemic glucose ranges (≥180 mg/dl) were 6.1%, 3.6% and 4.7%, respectively. Sensors indicated comparable performance on all days investigated (day 1, 3 and 5). None of the systems showed premature failures. In a porcine model, the performance of the biosensor revealed a promising performance. The transfer of these results into a human setting is the logical next step.

## Introduction

Hyperglycemia is common in critical illness irrespective of the presence of preexisting diabetes. Several theories have been raised claiming the activation of the hypothalamic-pituitary-adrenal-axis to be the main cause of so-called stress hyperglycemia, but also inflammatory processes, concomitant medication and acute renal impairments represent prominent triggers provoking hyperglycemia^1^. Hyperglycemia is a well-known risk factor for complications and increased mortality in both critically- and not critically ill medical and surgical patients^2–5^. Moreover, the degree of hyperglycemia has been associated with increased risk for unfavorable outcomes^6,7^.

Today, repetitive point-of-care measurements of capillary, venous or arterial blood samples in varying intervals is the recommended and established method to evaluate and steer glycemic control in the hospital both on the general ward and in critical care. These measurements are cheap and precise, but are associated with increased work-load of nursing staff, are – depending on blood-withdrawal method - associated with blood loss, can result in data transfer errors and only show glucose values in a low resolution without the availability of a continuous glucose profile.

Continuous glucose monitoring (CGM) systems which provide glucose measurements obtained from the interstitial fluid of subcutaneous tissue display glucose values in real time and have gained significant importance in the management of diabetes in the outpatient setting providing sufficient sensor accuracy for treatment decisions^8,9^. To date, various subcutaneous CGM systems were evaluated at general wards and in intensive care unit settings and were considered safe and effective for glucose monitoring in inpatients^10–14^. However, sensor performance data reported on subcutaneous CGM in critically ill vary and might depend on the patient population and sensor generation investigated^12,15^. In recent years not much research focused on subcutaneous CGM in critical illness and only one study reports accuracy data of a currently used and in terms of accuracy advanced CGM system^12^. There is still uncertainty regarding sensor accuracy in certain pathologic conditions (e.g. sepsis, hypovolemia, circulatory shock)^16^ and physiologic delays between interstitial and plasma glucose that might hinder the use of currently marketed subcutaneous CGM systems in critical care and consequently there is only little evidence available supporting potential benefits of these systems in critically ill patients^10,17^. Thus, no final conclusion on subcutaneous CGM in terms of accuracy and usefulness in criticall illness can be drawn at the moment.

To date, only few intravascular glucose monitoring techniques and devices – mainly using central venous and intraarterial access - have been tested and might overcome issues observed in subcutaneous glucose monotoring^18–21^. The few studies that directly compared the performance of intraarterial and subcutaneous CGM show conflicting results: one study showed superior performance of the intraarterial system whereas the other study showed comparable performance^22,23^. No direct comparison of central venous glucose monitoring and subcutaneous glucose monitoring is available to date.

Reported accuracy data on central venous catheter based continuous glucose monitoring varies largely between studies and use of different accuracy metrics make a comparison difficult^18,22,24^. It can be hypothesized that the superior performance of systems using the intravascular rather than the subcutaneous space can be attributed to the physiological lag-time between compartments also seen in non-critical illness but might be aggrieved due to alterations in subcutaneous microcirculation in critically illness and largely depend on tissue perfusion^15,25^. Although intravascular CGM systems are not affected by tissue lag time^26^ and sensor inaccuracy due to fluid retention, their use in clinical practice is still limited to four approved devices (GlucoClear by Edwards Life Sciences [Irvine, CA, USA]^27^, Glysure System by Glysure [Abingdon, Oxfordshire, UK]^20^, Eirus by Maquet Getinge Group [Rastatt, Germany]^28^ and Optiscanner 5000 by Optiscan [Hayward, CA, USA])^29^ in Europe^30^. In daily routine these systems have not yet reached broad clinical adoption as difficulties in regulatory approval, technical issues, side effects (thrombotic and infectious events in *in-vivo* devices) as well as unexpected but yet not clearly clarified drug interferences are factors resulting in a limited use^31^. It might be speculated that intravascular CGM combined with validated insulin titration protocols might in the future minimize glycemic variability, increase the time spent in glucose target, reduce workload and save costs^32,33^.

The aim of this pre-clinical *in-vivo* trial was to investigate the accuracy and safety of the SGC^PLUS^ intraarterial blood glucose measurement system, a system designed for arterial blood glucose monitoring in critical care, using hypo- and hyperglycemic clamp technique in anesthetized healthy pigs.

## Materials and methods

### Study overview

This was a monocentric, single-arm trial performed in six female anesthetized healthy farm pigs (*sus scrofa domestica*). The primary objective of this study was to investigate the accuracy of the BBraun arterial glucose measurement system compared to arterial blood glucose values.

The animal experiments were conducted meeting the requirements of the Austrian law and ethical regulations. The study was approved by the national committee for animal experiments (BMWF-66.010/0090-II/10b/2009 according to BGBl. Nr. 501/1989, i.d.F. BGBl. 1 Nr. 162/2005). The experiments were performed in accordance with the European Union (EU) legislation (EU Directive 2010/63/EU).

### B. Braun SGC^PLUS^ blood glucose measurement system

The B. Braun SGC^PLUS^ blood glucose measurement system includes a novel glucose sensor that can be integrated in the arterial line routinely used for invasive hemodynamic monitoring and arterial blood sampling.

**Figure 1:**
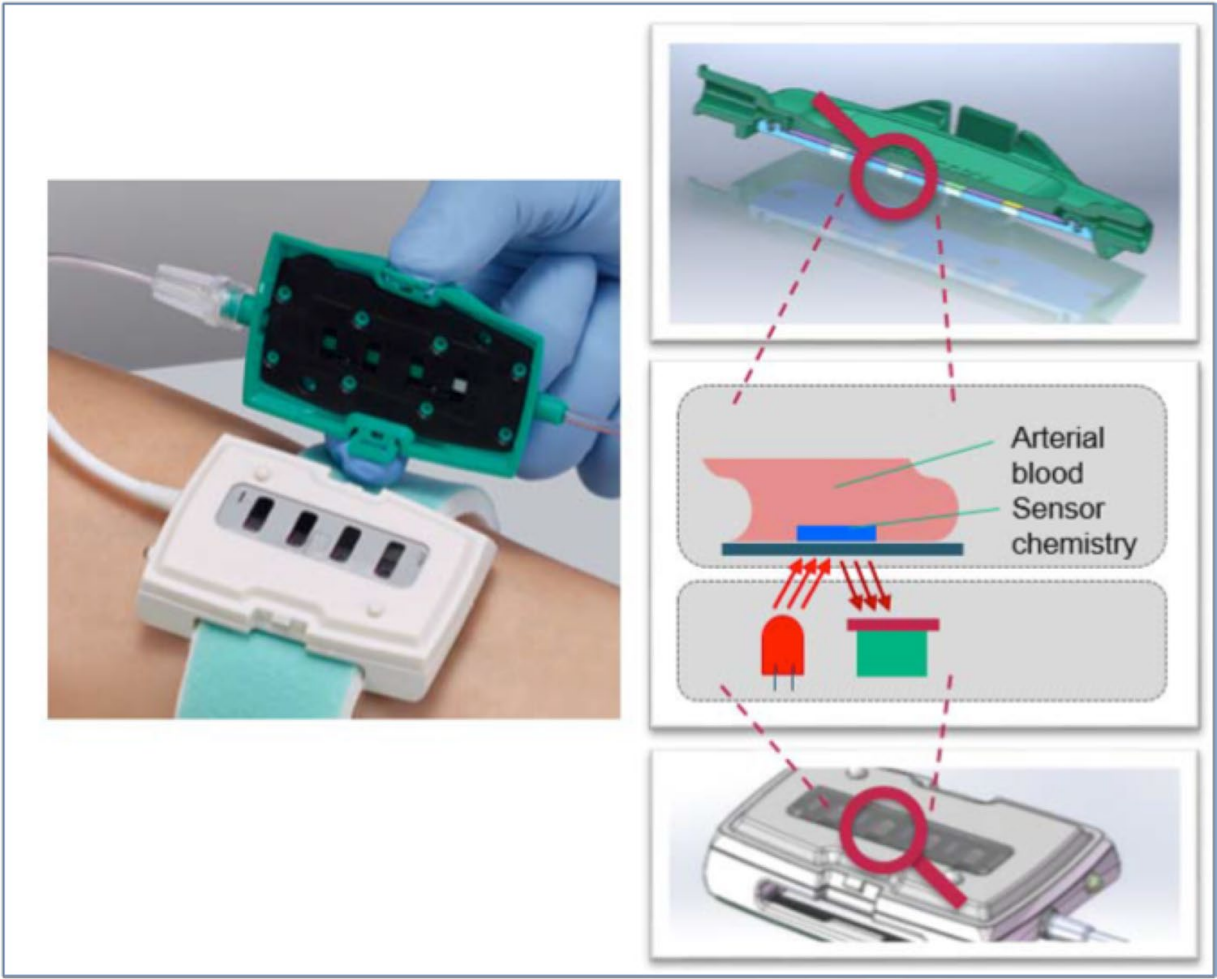
B. Braun SGC^PLUS^ blood glucose measurement system. The left picture indicates the reusable part of the sensor as attached to the patient arm (symbolic picture), responsible for optical activation and read-out of the sensor chemistry, and the disposable part (black/green) hosting the sensor chemistry and being connected to the arterial line. The right column of pictures indicates the principle functionality of the optical measurement technology as described in more detail in the document.

The sensor technology is based on optical fluorescence. Using this reversible technology, an optical indicator substance, specifically developed to be sensitive to a biomarker, is integrated into a disposable sensor as depicted below (Fig. 1). The indicator is activated using an external light source at a specific wavelength (620 nm). By the energy, transmitted as light impulse to the indicator, electrons achieve a higher state of energy which they immediately leave after the light impulse, and in return emit light at a different wavelength, which then can be mathematically processed to the biomarker concentration of interest. Glucose concentration is measured by analyzing the change of luminescence decay time during a certain measurement period of the oxygen concentration. This change is based upon the enzymatic reaction using glucose oxidase to reduce glucose to gluconolacton under consumption of oxygen. The applied sensor uses four measurement spots, two spots for glucose, one for oxygen and one for temperature measurement. Oxygen and temperature measurement are used for software compensation in order to improve the accuracy of the glucose reading. During the study, oxygen concentrations were in a physiological range within 125 and 180 mmHg.

In the current study setting, the disposable sensor was placed between the arterial catheter and the arterial line for hemodynamic monitoring and arterial blood sampling. To perform a blood glucose measurement, arterial blood is aspirated into the disposable sensor using a standard procedure as applied in the critical care setting. Once the system confirms that the measurement is completed, the blood is completely re-infused so that there is no blood loss in conjunction with the measurement procedure of this novel technology. During the present study, blood loss was solely caused by the required reference blood glucose determinations.

### Study procedures

On the first study day, 6 arterial lines were placed. Of these, 2 were inserted in each of the hind legs (*Arteria femoralis dextra and sinistra*) and one in each carotid artery. Next, the sensors were connected to the arterial lines and a continuous saline infusion was initiated in order to protect the system from clotting as the system was active during the full length of 5 consecutive days. One of the arterial lines was also used for collection of blood for system calibration and reference glucose measurements (Super GL Glucose Analyzer, Müller GmbH, Freital, Germany). Whereas separate studies (data not shown) indicated no adverse effect on arterial catheter maintenance, for reasons of hemodynamic stability of the animals, in the present study, low dose heparin (500 ml NaCl 0.9% + 10 ml heparin) was applied. Simultaneous aspirations and re-infusion of blood of each arterial line as well as withdrawal of blood for reference measurements were performed in 15-minute intervals. In order to avoid measurement delays, 3 persons from the trial staff were engaged for simultaneous sample collection of 2 sensors in parallel. After 10 hours of sampling (39 samples for each sensor), the sensors were placed in physiological saline solution (as used as standard arterial line maintenance solution) for 36 hours. On day 3 the same sensors as used for the experiment on day 1 were used for measurements in another anesthetized pig, again with a sample interval of 15 minutes for a total of 10 hours. This procedure was repeated again on day 5 in a third anesthetized animal. After the final 10 hours of sensor operation, this first block of experiments was finished and the sensors were deactivated.

The same experiment (from day 1 until day 5) was independently performed a second time, so that in total, the performance of 12 sensors in 6 pigs was available for analysis.

### Calibration procedure

Calibration of the sensor systems was performed using external reference glucose measurements determined by a blood gas analyzer. After a sensor initialization period of one hour, all sensors were calibrated using the corresponding arterial blood glucose measurement. The sensors were calibrated twice on each study day: the first calibration was performed using the first reference glucose measurement of each study day; 90 minutes after the first calibration, a second calibration was required. Thus, each sensor was calibrated six times during the study period of three days. Between first and second calibration, 5 measurements were performed. These measurements were included in the analysis.

### Anesthesia protocol

The pigs received premedication with intramuscularly 0.5 mg/kg midazolam (Midazolam, Nycomed, Austria), 2.5 mg/kg azaperone (Stresnil, Lilly Deutschland GmbH, Germany),10 mg/kg ketamine (Ketasol, aniMedica GmbH, Germany) and 0.2 mg/kg butorphanol (Butomidor, Richter Pharma AG, Austria). Fifteen minutes later, anesthesia was induced with intravenous propofol 1% (Diprivan, AstraZeneca, Austria) 3 mg/kg to effect and the pig was endotracheally intubated. The pigs were placed in dorsal recumbency and connected to an anesthetic circle system. Anesthesia was maintained with intravenous constant rate infusion (CRI) of propofol 1% (Diprivan, AstraZeneca, Austria) 2–5 mg/kg/h and fentanyl (Fentanyl-Janssen, Janssen-Cilag Pharma GmbH, Austria) 20 μg/kg/h and optionally inhalation anesthesia with isoflurane (Forane, Abbott, Germany) 1–2% was used to deepen general anesthesia.

For mechanical ventilation of the lungs, IPPV (intermittent positive pressure ventilation) was used with an Inspiration: Expiration ratio of 1:2, a tidal volume of 10–15 ml/kg, a respiratory rate between 12–20/min and a PEEP (positive end expiratory pressure) of 5 cm H_2_O, to keep the endtidal CO_2_ within physiologic limits. All pigs received an intravenous infusion of 10 ml/kg of Elo-Mel Isoton (Fresenius-Kabi, Austria).

Clinical monitoring was performed using an anesthetic monitor. Parameters measured included: respiratory rate derived from the capnogram, invasive mean arterial blood pressure, respiratory gas composition including inspiratory and expiratory concentrations of O_2_, CO_2_, and isoflurane, pulse oximetry and oxygen saturation and core body temperature using an esophageal probe.

At the end of experiment all animals were euthanized in deep general anesthesia using an intravenous overdose of potassium chloride.

### Clamp protocol

**Figure 2:**
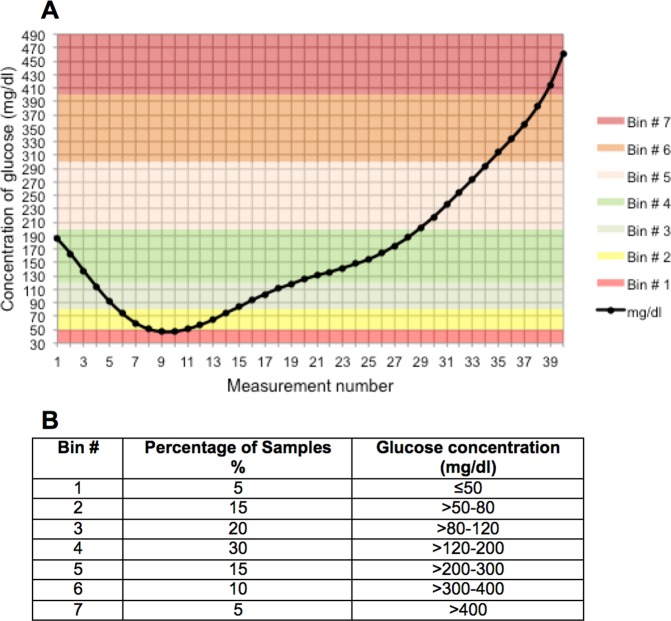
A Glucose clamp scheme for each study day. The Bins are illustrating different glucose areas *B* Percentual distribution of samples according to ISO 15197:2013.

The ISO15197:2013 suggests to investigate a broad range of hypo, eu- and hyperglycemic glucose values. To achieve this goal during all study days, the same glucose clamp procedure was conducted in all experiments. The glucose clamping scheme as well as the suggested percentage of clamped glucose values within prespecified glucose target areas according to ISO15197:2013 is illustrated in Fig. 2.

### Data analysis

For data analysis, sensor glucose values were matched with the corresponding arterial glucose values. Overall sensor accuracy was determined using ISO 15197:2013 (percentage of sensor values that are within ±15 mg/dl of the reference value at glucose concentrations <100 mg/dl and within ±15% at glucose concentrations ≥100 mg/dl). Additionally, the CLSI (Clinical Laboratory Standards Institute) POCT12-A3 Standard, a guideline to assess point-of-care glucose meter performance in the hospital, was used^34,35^. Overall accuracy as well as accuracy during hypoglycemia (<70 mg/dl), euglycemia (≥70–180 mg/dl) and hyperglycemia (≥180 mg/dl) was determined by calculating the mean absolute relative difference between the measurements provided by the sensor and arterial reference. Arterial sensor values and reference glucose values are displayed as Bland-Altman plot and Parkes Error Grid analysis.

### Primary endpoint

The primary endpoint of the experiment was to assess the achievement of the accuracy criterion described in ISO 15197:2013, indicating that at least 95% of the measurements differ from reference glucose by a maximum of 15 mg/dl for glucose concentrations <100 mg/dl, and by a maximum of 15% for glucose concentrations ≥100 mg/dl.

### Sample size calculation

As this was a feasibility trial, no formal sample size calculation was performed.

## Results

All experiments were performed as planned; the average relative error to achieve the targeted glucose values within the clamp experiments was low (3.2%). None of the sensors reported any premature failure or malfunction during the observational period. Furthermore, all sensors were used over the dedicated run-time (96 hours) without revealing any sensor failure or relevant measurement deviation after a total of 107 hours of use. No hard- or software had to be repaired or replaced during the entire trial.

Sensor performance was tested among a wide range of blood glucose values (test range: 37–458 mg/dl). Of 1404 scheduled measurements, 1337 (95.2%) pairs were available for analysis at the end of the last study day. 67 readings were not included in the analysis as they were used for sensor calibration. 18 sensor readings were not available due to arterial line failure or unsuccessful aspirations (1.2%).

Within the entire glycemic range (37–458 mg/dl), the sensors met ISO15197:2013 criteria in 99.2%. For prespecified glucose areas as defined within ISO 15197:2013 the percentages of values meeting the criterion were as follows: <100 mg/dl (n = 414) 100%, and ≥100 mg/dl (n = 923) 98.8%. Further, even more stringent deviations are indicated in Table 1.

**Table 1:** %deviation from sensor to reference glucose (overall and split for values <100 mg/dl and ≥100 mg/dl).

% deviation from reference	<100 mg/dl (n = 414)	≥100 mg/dl (n = 923)	All (n = 1337)
<15%	100% (414)	98.8% (912)	99.2% (1326)
<10%	99.0% (410)	91.5% (845)	93.8% (1255)
<5%	79.2% (328)	58.5% (540)	64.9% (868)

The CLSI POCT-12-A3 standard requires that 95% of the results must differ from the reference system less than ±12 mg/dl at glucose concentrations <100 mg/dl and less than ±12.5% at glucose concentrations ≥100 mg/dl (criterion 1); the sum of the number of individual results with errors that exceed ±15 mg/dl at glucose concentrations <75 mg/dl and exceed ±20% at glucose concentrations ≥75 mg/dl should not exceed 2% of all measurements (criertion 2)^34,35^. In our study 97.5% of sensor values fulfilled criterion 1 and only 0.4% exceeded the boundaries set in criterion 2. Eleven out of 923 sensor values differed more than 15% from reference with a maximum deviation of 18.2%. All of these deviations occurred at glucose levels ≥300 mg/dl.

Mean absolute relative difference (MARD) during the entire glycemic range of all sensors was 4.3%. The MARD within the hypoglycemic (<70 mg/dl), euglycemic (≥70–180 mg/dl) and hyperglycemic ranges (≥180 mg/dl) were 6.1%, 3.6% and 4.7%, respectively. Comparing MARDs regarding the time of use (days 1, 3 and 5) sensor accuracy showed to perform best during day 1 but exhibited worst results in hypoglycemia. Individual sensor performance data and sensor performance in different glycemic ranges are displayed in Table 2A,B.

**Table 2:** **A** Mean Absolute Relative Differences (MARDs, n = number of available value pairs) for different glycemic ranges. **B** MARDs on days 1, 3 and 5 of sensor use.

A				
Sensor	Overall (n)	Hypoglycemia < 70 mg/dl (n)	Euglycemia ≥ 70–180 mg/dl (n)	Hyperglycemia ≥ 180 mg/dl (n)
Sensor 1	4.3 (111)	6.1 (21)	3.2 (49)	5.2 (41)
Sensor 2	5.2 (110)	6.4 (21)	4.5 (49)	5.1 (40)
Sensor 3	2.5 (113)	4.5 (21)	2.2 (49)	2.5 (43)
Sensor 4	5.1 (112)	4.3 (21)	5.0 (49)	5.7 (42)
Sensor 5	6.4 (112)	5.3 (21)	6.8 (49)	7.0 (42)
Sensor 6	3.6 (112)	3.9 (21)	3.4 (47)	3.6 (44)
Sensor 7	5.0 (108)	5.3 (19)	5.1 (50)	4.8 (39)
Sensor 8	5.1 (111)	8.6 (19)	3.1 (51)	5.5 (41)
Sensor 9	3.4 (112)	4.7 (19)	3.2 (52)	3.5 (41)
Sensor 10	3.5 (113)	9.6 (19)	2.8 (52)	3.7 (42)
Sensor 11	5.5 (110)	8.9 (19)	5.6 (52)	4.5 (39)
Sensor 12	3.4 (113)	6.0 (19)	3.1 (52)	3.3 (42)
All Sensors	4.3 (1337)	6.1 (240)	3.6 (601)	4.7 (496)
**B**				
**Days of use**	**Overall (n)**	**Hypoglycemia (n)**	**Euglycemia (n)**	**Hyperglycemia (n)**
Day 1	3.5 (424)	10.5 (78)	2.8 (191)	3.1 (155)
Day 3	4.7 (456)	4.3 (84)	4.6 (209)	5.2 (163)
Day 5	4.6 (457)	5.0 (78)	3.3 (201)	6.1 (178)

**Figure 3:**
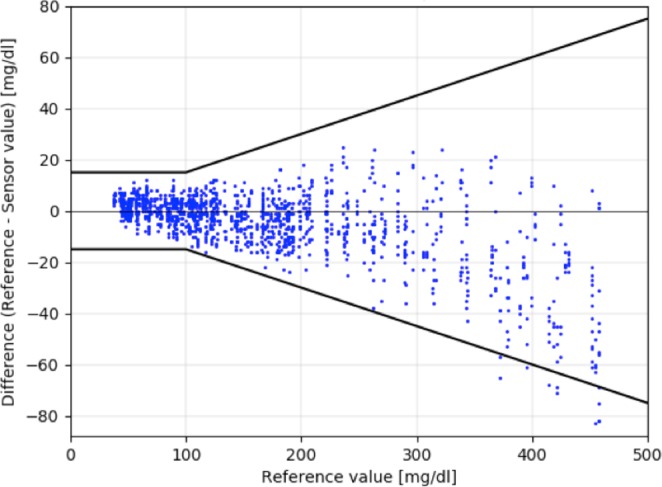
1337 sensor-reference pairs displayed in the adjusted Bland-Altman plot including indication of fulfilment of ISO 15197:2013 criteria (15 mg/dl for glucose values <100 mg/dl and 15% for glucose values ≥100 mg/dl, bold black line). On the x-axis the reference glucose values are indicated. On the y-axis the deviation in mg/dl from sensor of 1337 sensor-reference pairs.

The Bland-Altman plot (Fig. 3) displays the distribution of sensor values and their deviations from reference within the whole range of glycemia.

**Figure 4:**
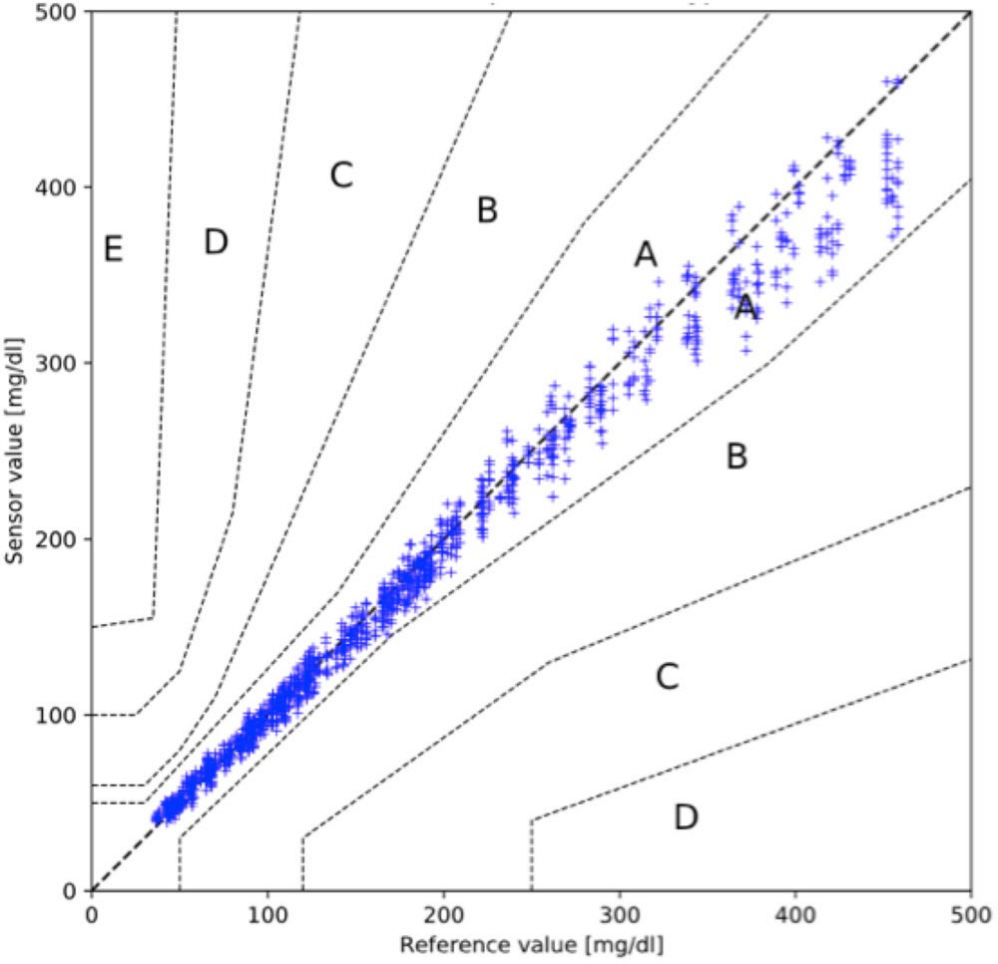
Parkes Error Grid for 1337 sensor-reference value pairs: On the y-axis sensor glucose is displayed, on the x-axis reference glucose is indicated. The grid is divided into zones displaying the degree of potential risk caused by erroneous measurements: values in zone *A* do not alter clinical action; zone *B* indicates altered clinical action with small or no significant effect on clinical outcome; zone *C* shows altered clinical action with probable effect on clinical outcome; zone *D* results in altered clinical action which could be associated with significant medical risk; and zone *E* causes altered clinical action which could have dangerous consequences.

When assessing the overall clinical sensor performance using the Parkes Error Grid (Fig. 4), 100% of sensor–reference glucose pairs were situated in Zone A (benign error) which would not result in erroneous and potentially harmful treatment decisions.

## Discussion

This study evaluated the accuracy of a novel glucose sensor for arterial blood glucose measurement in a clamp study in pigs. The sensor was able to accurately measure arterial glucose concentrations meeting the ISO 15197:2013 criteria in 99.2% of all measured values. The sensors also fulfilled the more stringent requirements of the CLSI POCT-12-A3 standard with 97.5% of sensor values fulfilling criterion 1 and only 0.4% exceeding the boundaries of criterion 2. The overall MARD was low (4.3%); 7 out of the 12 sensors tested exhibitted a MARD of <5% and all sensors remained with their total MARDs below 6.5%. Of note, the sensors showed to be most accurate in euglycemia (MARD 3.6%) and on the first day of use (MARD 3.5%).

Also, clinical accuracy assessed by Parkes Error Grid was high (100% of all values located in zone A). No early sensor failure occurred. With these accuracy results the novel vascular glucose sensor outperformed currently available subcutaneous glucose monitoring systems both in diabetes outpatient care as well as in critical care.

In contrast to measuring interstitial glucose concentrations in otherwise healthy people with diabetes, sensor performance might differ in critical illness e.g. due to altered tissue perfusion in sepsis^15,36^. Thus, intravenous or intraarterial glucose sensing might be a more reliable alternative as vascular access is granted in the majority of critically ill patients. Intravascular glucose monitoring data is still scarce.

A head-to-head comparison of two different CGM systems – intravascular microdialysis vs. subcutaneous glucose monitoring - was performed by Schierenbeck at al. in patients undergoing cardiac surgery^22^. Neither of the systems tested met ISO 15197:2013 criteria, which in contrast were met by 99.2% of sensor-reference pairs in our study. MARD was 6.5% for intravascular microdialysis and 30.5% for subcutaneous glucose monitoring. These results indicate that the vascular approach might be more accurate in certain conditions of critical illness and the results were comparable to results obtained in our trial (MARD 4.3%).

A prospective study assessing the performance of a continuous intravenous microdialysis-based glucose monitoring device showed good sensor accuracy when tested in 12 critically ill patients^37^. When comparing the results of our study with the results reported by Leopold *et al*. sensor performance of the novel sensor was slightly superior (MARD 4.3% vs. 7.5%; 100% of values in zone A of Parks vs. 93.6% of values in zone A of Clarke Error Grid). No early sensor failure occurred in our study whereas 4 out of 12 sensors needed to be replaced in the depicted study by Leopold *et al*.

Our data, in line with other data reported on performance of vascular glucose monitoring even outperform the MARD of 10%, which is requested in diabetes care^38^ for non-adjunct continuous subcutaneous glucose monitoring and easily meets criteria suggested for critical care (MARD < 14%)^39^.

Similar to the monitoring of vital signs as blood pressure, respiratory information or body temperature, also the actual glycemic value should be displayed on the monitor screen beside the patient´s bed. Firstly, this real-time glucose measurement might help to give health care professionals safety and encouragement to adjust glycemic control more intensively. Secondly, frequent glucose profiling is time consuming and CGM systems in the hospital might economize the time expenses for nursing and laboratory staff^40^. Third, reliable and standardized CGM systems might pave the way for the implementation of semiclosed/closed loop systems for inpatients – these in turn might serve as tools for the establishment of properly achieved tight glycemic control in the hospital^41^.

In contrast to subcutaneously placed glucose monitoring systems measuring interstitial blood glucose, to date, intravascular systems have remained behind the stage for the use in clinical practice^31^. Both *in-vivo* and *ex-vivo* systems, employing different access sites and providing various sampling and detection methods, have been tested by various companies so far. However, only a few systems have been approved by regulators so far^30,42^. Despite promising results concerning accuracy, mainly the need for intravascular access of these systems and its “foreign-body” response potentially causing thrombus formation, infection and vessel injury, as well as technical, regulatory and usability concerns have been limiting factors against the implementation of such systems in daily routine^31,43,44^. These concerns can be eliminated by the fact that the system does not require a direct connection to the vascular system as it is linked extracorporally as interponate in between the tubes of the conventionally installed arterial line which ICU patients require anyway for hemodynamic monitoring and blood sampling, e.g. for blood gas checks. Therefore, sensor-related vascular complications (thrombosis, infection, vessel injury) can be neglected.

Another major aspect for the suitability of the technology in the critical care setting is the fact that using the technology, the aspirated blood that is required for glucose analysis is completely reinfused into the organism, such as that using this technology, blood loss due to glycemic management can be completely omitted.

The strength of this study is that it was performed under well-controlled conditions in deeply anesthetized swine. By performing the glucose clamp procedure, it was granted that the sensor was tested over a broad range of glycemia (37–458 mg/dl). Under routine clinical conditions the number of hypoglycemic episodes is low. Due to our clamp design we managed to evaluate sensor performance also in hypoglycemia with a substantial number of glucose values in the hypoglycemic range (240 sensor pairs); we were able to proof excellent sensor performance also in this critical range. Another strength of the study is that sensor performance was tested on different days of sensor use.

One limitation of the study is that it was performed in a relatively small sample size and in an animal model. Due to the large number of sensor-reference pairs (n = 1337) this risk was mitigated. As it was the first use of the sensor in living individuals, it was required to perform an animal study before moving to critically ill patients. As arterial access is required for the sensor set-up it is unethical to perform such a test in healthy volunteers. A further limiting factor is that the sensors were not exposed to movement or other influencing environmental factors such as temperature change or acute derailments of health status, fever or co-medication. However, the latter mentioned (potential interaction of co-medication such as vasopressors or acetaminophen) has not been yet explicitly investigated or reported in studies which investigated intravascular glucose monitoring systems and needs further attention. Additionally, it needs to be acknowledged that a sensor was not continuously tested in one individual over the study period of 5 consecutive days. This limitation is due to the set-up of the experiment in pigs who do not tolerate anesthesia for such a long period.

Further studies are needed to confirm the sensor performance in different groups of critically ill patients.

## Conclusions

The current study confirmed that the novel sensor designed for glucose monitoring in arterial blood exhibits good performance and glucose data derived from that technology is accurate and reliable. These promising results gained from an animal study pave the way for changing glucose monitoring in clinical care.
